# Reproductive psychiatric advance directives: promoting autonomy for perinatal people with serious mental illness diagnoses

**DOI:** 10.1007/s00737-023-01382-5

**Published:** 2023-11-10

**Authors:** Emily C. Dossett, Sonja L. Castañeda-Cudney, Michelle T. Nguyen, Melisa Olgun, Jennifer Wang, Keris Jän Myrick, Laurie Hallmark, Elyn R. Saks

**Affiliations:** 1 Departments of Psychiatry and Behavioral Sciences and of Obstetrics and Gynecology, Keck School of Medicine, University of Southern California, 2250 Alcazar Street, Suite 2200, Los Angeles, CA 90033 USA; 2 Price School of Policy, University of Southern California, Los Angeles, CA USA; 3 Division of Maternal-Fetal Medicine, Department of Obstetrics and Gynecology, Keck School of Medicine, University of Southern California, Los Angeles, CA USA; 4 Yale School of Law, Yale University, New Haven, CT USA; 5 Keck School of Medicine, University of Southern California, Los Angeles, CA USA; 6 Disability Rights California, Los Angeles, CA USA; 7 Attorney and Subject Matter Expert Consultant Psychiatric Advance Directives, The Hallmark Compass, LLC, Sacramento, CA USA; 8 Gould School of Law, University of Southern California, Los Angeles, CA USA

**Keywords:** Psychiatric advance directive, Autonomy, Perinatal mental health, Pregnancy, Serious mental illness

## Abstract

People with serious mental illness (SMI) diagnoses who become pregnant are particularly vulnerable to symptom recurrence and resulting potential lack of decision-making capacity (Taylor et al. J Psychiatr Res 104:100-107, 2018; Bagadia et al. Int J Soc Psychiatry 66:792-798, 2020). In these situations, prenatal and behavioral health providers have little legally viable guidance on what medical and/or psychiatric care the patient desires (Aneja and Arora Indian J Med Ethics V:133-139, 2020). We created a “Reproductive Psychiatric Advance Directive (PAD),” grounded in Reproductive Justice principles, that promotes patient autonomy by proactively articulating perinatal medical and psychiatric care preferences. We conducted a medical and legal literature review using two sets of terms related to (1) PADs and (2) reproductive health. We convened an expert working group of legal, medical, psychiatric, peer, and advocacy leaders and community-based organizations to develop a Reproductive PAD. Our literature review yielded no results about Reproductive PADs. We created de novo a Reproductive PAD template with sections on medical and psychiatric history, informed consent for critical medical and psychiatric care, family planning and custody preferences, and optional sections on abortion and on electroconvulsive therapy. The Reproductive PAD provides a possible legal mechanism for people of childbearing age with SMI diagnoses to articulate their medical and psychiatric care choices around reproduction and pregnancy. Future research should evaluate the Reproductive PAD as an effective tool for protecting patient autonomy during pregnancy and postpartum and guiding medical and psychiatric providers.

**Supplementary Information:**

The online version contains supplementary material available at 10.1007/s00737-023-01382-5.

## Introduction

Perinatal people with serious mental illness (SMI) diagnoses have historically been neglected in both the fields of mental health and reproductive health care, with disastrous—though often preventable—outcomes for parents, pregnancies, infants, families, and communities (Meltzer-Brody and Stuebe 2014). Perinatal suicide ranks as one of the leading causes of pregnancy-related death, even though the Centers of Disease Control deemed 100% of previous cases preventable (Trost et al. 2021). Postpartum psychosis is accompanied by infanticide and suicide rates of 4% and 5%, respectively, and the illness itself recurs in 30% of subsequent pregnancies without prevention (Bergink et al. 2016). Despite the preventability of both these and other adverse outcomes, people with SMI often fall out of psychiatric care when they become pregnant, with resulting relapse of symptoms and mental health crises (Cox et al. 2016; Dolman et al. 2013; Viguera et al. 2007). Our goals in creating a Reproductive Psychiatric Advance Directive template are to improve perinatal psychiatric and medical care, reduce these preventable adverse outcomes, preserve autonomy and dignity in decision-making, and promote Reproductive Justice for perinatal people with SMI diagnoses.

## Background

“Reproductive Justice” combines reproductive rights and social justice into one movement with three pillars: first, everyone has the right to have a child; second, everyone has the right *not* to have a child; and third, everyone has the right to raise a child in a safe, healthy environment (Ross et al. 2017). The concept of Reproductive Justice was articulated in Chicago in 1994 by the Women of African Descent for Reproductive Justice in an effort to include Black voices in the women’s rights movement and expand the reproductive rights conversation beyond an exclusive focus on abortion access. Since then, the Reproductive Justice movement has grown to include a wide coalition of marginalized communities, including Black, LatinX, Asian American/Pacific Islander (AAPI), LGBTQ + , and people with disabilities (Sister Song n.d.), among others. One notable exception is people with SMI diagnoses, even though they have experienced the violation of all three pillars of Reproductive Justice throughout our country’s history.

The right for a person with mental illness to have a child was violated in 1927 in the now-infamous Supreme Court *Buck v. Bell* trial, which forced a woman inaccurately deemed “feeble-minded” to undergo a hysterectomy. In establishing that society “can prevent those who are manifestly unfit from continuing their kind,” the Court upheld the constitutionality of sterilization for the mentally disabled (Buck v. Bell 1927). While subsequent law has largely mitigated this ruling, it has never been formally overturned (McIntyre 2007).

The right NOT to have a child is most glaringly violated by the recent *Dobbs v. Jackson Women’s Health Organization* Supreme Court decision that overturned 40 years of abortion access. For people with SMI diagnoses, denial of abortion services is particularly problematic because of the lack of access to basic contraception and reproductive health care, lower health literacy, and higher rates of unintended or coerced sex (Miller 1997; Miller and Finnerty 1998). Statistics bear this out: while the U.S. rate of unintended pregnancy in the general population hovers around 45%, it is a striking 65% for women with SMI diagnoses (Schonewille et al. 2022).

Finally, the right to raise a child in a healthy, safe environment is constantly threatened for parents with SMI diagnoses. Child Protective Services (CPS) agencies frequently open cases on families simply because of a mental health diagnosis, even when no other red flags are evident (O’Donnell et al. 2015). This is especially true for Black, Indigenous, and People of Color (BIPOC) communities (Hammond et al. 2017). Even when CPS is not involved, parents with mental illness often face significant stigma that contributes to poverty, under-employment or unemployment, and a lack of social networks and support that make child-rearing precarious (Dolman et al. 2013).

Our approach to addressing these injustices for people of childbearing age with SMI diagnoses was to develop a template for Psychiatric Advance Directives (PADs) focused on reproductive health and care choices. In general, a PAD is a written document that provides individuals with SMI diagnoses the opportunity to articulate treatment preferences in advance, during times of stability (Murray and Wortzel 2019). The PAD can then be used during psychiatric crises or when the individual is deemed without capacity to make medical decisions to ensure their treatment choices are known and hopefully respected. A PAD works to protect autonomy and self-determination for people with SMI diagnoses, as well as to advocate for their psychiatric, medical, and social assistance choices. PADs have been in existence for years and implemented in several states (Swanson et al. 2006).Our home state, California, is currently funding a 4-year initiative to develop, launch, train, and evaluate a PAD in five pilot counties, followed by proposed legislation to implement it statewide (Ca.gov n.d.).

However, reproductive choices—including family planning, pregnancy continuation versus termination, perinatal mental health care, labor and delivery options, and custody planning—have not been developed for inclusion within a PAD. Our expert working group aimed to develop a “Reproductive PAD” to remedy this gap, highlight the reproductive needs of individuals with SMI diagnoses, and prevent adverse and potentially fatal outcomes, including maternal suicide and infanticide. An additional goal of developing the Reproductive PAD will be its inclusion in California’s larger PAD initiative.

## Methods

### Expert working group and community partners

An expert working group was formed and consisted of (1) a reproductive psychiatrist with extensive policy background; (2) an attorney with lived experience and academic and research expertise in mental health law, policy, and ethics; (3) a policy expert with lived experience with perinatal mental illness; (4) a maternal–fetal medicine physician with expertise in medical ethics; (5) an attorney and PAD subject matter expert with lived experience; (6) a subject matter expert on peer specialists and training; and (7) two trainees, one in law school and one in medical school. The expert working group also developed a working collaboration with two different peer community-based organizations. The first was Painted Brain, a community-based organization that is run by and for peers with SMI diagnoses and promotes recovery through arts, advocacy, and skills training. Painted Brain is the peer organization tapped by California to help develop the general PAD as part of its statewide initiative. The second was Disability Rights California (DRC), an advocacy group focused on defending and strengthening the rights of people with disabilities. Specifically, we worked with their Peer Self-Advocacy staff, which included several members with lived experience.

### Literature review

We conducted a broad literature review on reproductive decision-making as part of Psychiatric Advance Directives, given the novelty and specificity of the topic. For the literature review, group members used databases from both medicine and law: Pubmed, Medline, Proquest, and Hein Online. There were no location, language, or date restrictions, as we hypothesized that there would be a minimal amount of existing research and we wanted to cast the widest net possible. For every search, we used at least one term in each of two categories. The first category explored PADs, and the following search terms were used: “psychiatric + advance + directives,” “values + based + decision-making,” “conservatorship,” and “serious + mental + illness.” The second category was reproductive or perinatal health, choice, or planning, and the following terms were used: “pregnancy,” “childbirth,” “contraception,” “family + planning,” and “perinatal + mental + health.” For instance, search terms used in combination included “psychiatric + advance + directives” and “contraception.” This process took place from May 2022 to March 2023. Institutional Review Board approval was not required since no patient data was accessed or utilized.

After initial records were identified, they were narrowed down based on relevancy and robustness of information. Screening was performed by abstract review; this was done by three team members (ECD, JW, and MO). Inclusion criteria consisted of whether or not the abstract discussed both Psychiatric Advance Directives (directly or in theory) and reproductive or perinatal planning, choice, or decision-making. Any articles about PADs or that contained search terms were read in full. Disagreements about inclusion were discussed in research team meetings until a consensus was reached.

### Template development

After the literature review, regular team meetings were held to create the Reproductive PAD template. We utilized a spreadsheet, created by our subject matter expert on PADs, to organize our template draft in alignment with the larger California project. We created subcategories of relevant topics and then potential questions or areas of education within each one. These subcategories and questions were developed through consensus at research team meetings, with each member contributing, editing, and refining according to their subject matter expertise. Any disagreements were resolved through consensus.

Once an acceptable preliminary draft was complete, Painted Brain was presented to their internal working group for feedback. The director then returned all comments and suggestions back to our research team. Next, the Disability Rights California peer self-advocacy group held an online feedback session for any interested staff or peers. The principal investigator (ECD) attended and brought notes on the template back to the expert working group. We utilized this community-based feedback to further refine and finalize the Reproductive PAD template.

## Results

### Literature review

One hundred forty-one potential articles were located in medical and law databases. Methodologies included qualitative studies, cross-sectional survey studies, longitudinal studies, informational articles, and trial studies. We did not exclude any articles based on field (law versus medicine) or methodology. All articles were in English or had been translated at publication from French into English.

**Fig. 1:**
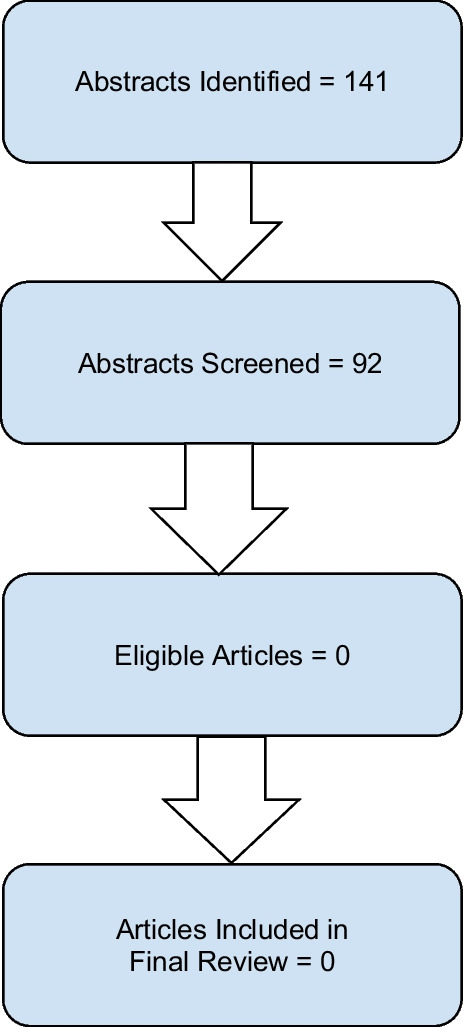
Literature review results

After reviewing abstracts and removing results that did not include a search team from both the PAD/decision-making and the reproductive health categories, 92 results remained. These articles were divided among the research team for further review, with the goal of determining if any of them investigated both Psychiatric Advance Directives and any sort of reproductive health, planning, or choice topic. No articles were found that included both. This verified our hypothesis that this work is novel (Fig. 1).

### Template

The final Reproductive PAD template contains questions that must be answered to safely manage pregnancy, labor, delivery, and postpartum care for a perinatal person during times of psychiatric crisis. The premise is that a person with an SMI diagnosis has the capacity at the time of completing the PAD to make these decisions but understands that the document will guide their care at a future time when that capacity may be impaired. Our standard for including a question was whether or not the decision (1) could be answered in advance, (2) was legally allowable to be made by a surrogate decision-making or agent, and (3) was vital to safe medical and psychiatric care in the moment of crisis.

The final template contains three primary sections: information on medical history and previous pregnancies that are crucial for the prenatal care provider; preferences and informed consent for key medical and psychiatric treatment; and preferences for essential reproductive health and parenting choices. The language is designed to be accessible enough for a person to fill it out independently, though ideally a peer or provider is able to assist (see Appendix A for Reproductive PAD template).

In addition, we created two additional “optional” sections. The first section reviews options for pregnancy continuation versus termination. Legally, a surrogate decision-making or health care agent cannot choose to end a pregnancy for a person unable to make that decision for themselves, even if designated in an advance directive. By this reading of the law, a person lacking capacity due to psychiatric crisis cannot undergo an abortion, even if they have made their desire for one clear in the past. This is true even in states, like California, where abortion remains legal; many other states have very limited access to legal abortions in any circumstance. Despite this, we are keeping the section as “optional” in order that the person filling out the Reproductive PAD can reflect on this issue and clarify desires and values regarding pregnancy continuation versus termination in non-crisis situations.

The second optional section asks about preferences regarding electroconvulsive therapy (ECT). ECT remains one of the most highly effective treatments that we have for treatment resistant mood and psychotic disorders, and the safety profile in pregnancy is reassuring (Ward et al 2018). However, ECT remains highly stigmatized, and members of our expert working group with lived experience advised that including it in the main body of the Reproductive PAD could pose more of a barrier than a help. In addition, many people do not have access to ECT, particularly if they are on public insurance or lack capacity to consent to it. We only wanted to offer choices in the Reproductive PAD that were feasible. For both of these reasons, we moved questions about ECT preferences into an “optional” section, for those who are open to, and able to access, ECT.

## Discussion and impact

In the realms of mental health and reproductive choice, perinatal people with SMI diagnoses have a unique set of needs and risks that require innovative approaches. However, because these two realms rarely overlap, we were unable to find any legally viable mechanisms that would support a person of childbearing age with a serious mental illness diagnosis in documenting their own reproductive preferences and plans. Per the tenets of Reproductive Justice, however, this population has the same rights as anyone else to have a child, to not have a child, and to raise a child in a healthy, safe environment. We believe that the novel Reproductive PAD can promote these rights.

At the same time, we understand through lived experience and clinical expertise that safe parenting may not be feasible for all individuals with mental health challenges, all of the time. This is particularly true in the United States, where arcane convoluted rules around access health care and public benefits, punitive child welfare systems, and stigma against the mentally ill—particularly those who are also BIPOC or otherwise marginalized—throw up more barriers to supported parenting than assistance. In certain circumstances, the right to raise a child may need to be revoked, either temporarily or permanently, if the parent cannot provide safety. Even in these situations, however, the birthing person should be able to articulate a preference as to who does parent their child, and we included specifications for custody as a key area in the Reproductive PAD.

Strengths of this study include the varying and extensive subject matter and lived expertise of different working group members. We incorporated medical, legal, advocacy, ethics, and educational perspectives that ranged from decades of experience to current trainees. An additional strength is the collaboration with two community-based organizations to gather feedback from a wide range of individuals, including peers for disability rights and mental health advocates across rural and urban regions. This range of expertise is essential because there is little existing literature about PADs in general or reproductive health in perinatal people with SMI diagnoses, and none on Reproductive PADs.

At this point, the primary limitation of our work is its novelty and the need to implement it in clinical populations for study. We designed the Reproductive PAD to be a living document, open to continuous shaping and refinement through clinical application. We are hopeful that for individuals with SMI diagnoses, the Reproductive PAD will be an innovative and effective tool for spotlighting reproductive health needs and desires, promoting autonomy, advocating for medical and psychiatric care preferences, and preventing adverse and even fatal perinatal outcomes.

## Supplementary Information

Below is the link to the electronic supplementary material.

Supplementary file1 (ZIP 26 KB)

## References

[CR1] Aneja J, Arora S (2020) Pregnancy and severe mental illness: confounding ethical doctrines. Indian J Med Ethics V:133–139. 10.20529/IJME.2020.037 32393450 10.20529/IJME.2020.037

[CR2] Bagadia A, Nanjundaswamy M, Ganjekar S, Thippeswamy H, Desai G, Chandra PS (2020) Factors influencing decision-making around pregnancy among women with severe mental illness (SM): a qualitative study. Int J Soc Psychiatry 66:792–798. 10.1177/0020764030925104 32579050 10.1177/0020764020925104

[CR3] Bergink V, Rasgon N, Wisner KL (2016) Postpartum psychosis: madness, mania, and melancholia in motherhood. Am J Psychiatry 173:1179–1188. 10.1176/appi.ajp.2016.16040454 27609245 10.1176/appi.ajp.2016.16040454

[CR4] Buck v. Bell, 274 U.S. 200, 207 (1927)

[CR5] CA.gov (n.d.) Psychiatric Advance Directives- MULTI-COUNTY COLLABORATIVE, Mental Health Services Act Innovations Project. CA.gov. Retrieved April 27, 2023, from https://mhsoac.ca.gov/sites/default/files/Multi%20County_INN_PADs_0.pdf

[CR6] Cox EQ, Sowa NA, Meltzer-Brody SE, Gaynes BN (2016) The perinatal depression treatment cascade: baby steps toward improving outcomes. J Clin Psychiatry 77:1189–1200 27780317 10.4088/JCP.15r10174

[CR7] Dolman C, Jones I, Howard LM (2013) Preconception to parenting: a systematic review and meta-synthesis of the qualitative literature on motherhood for women with severe mental illness. Arch Women Mental Health 16:173–196. 10.1007/s00737-013-0336-0 10.1007/s00737-013-0336-0 23525788

[CR8] Hammond I, Eastman AL, Leventhal JM, Putnam-Hornstein E (2017) Maternal mental health disorders and reports to child protective services: a birth cohort study. Int J Env Res Public Health 14:1320. 10.3390/ijerph14111320 29084185 10.3390/ijerph14111320 PMC5707959

[CR9] McIntyre M (2007) Buck v. Bell and beyond: a revisited standard to evaluate the best interests of the mentally disabled in the sterilization context. U Ill l Rev 1303:1311–1312

[CR10] Meltzer-Brody S, Stuebe A (2014) The long-term psychiatric and medical prognosis of perinatal mental illness: best practice and research. Clin Obstet Gynecol 28:49–60. 10.1016/j.bpobgyn.2013.08.009 10.1016/j.bpobgyn.2013.08.009 PMC3947371 24063973

[CR11] Miller LJ (1997) Sexuality, reproduction, and family planning in women with schizophrenia. Schizophr Bull 23:623–635. 10.1093/schbul/23.4.623 9365999 10.1093/schbul/23.4.623

[CR12] Miller LJ, Finnerty M (1998) Family planning knowledge, attitudes and practices in women with schizophrenic spectrum disorders. J Psychosom Obstet Gynaecol 19:210–217. 10.3109/01674829809025699 9929847 10.3109/01674829809025699

[CR13] Murray H, Wortzel HS (2019) Psychiatric advance directives: origins, benefits, challenges, and future directions. J Psych Prac 25:303–307. 10.1097/PRA.0000000000000401 10.1097/PRA.0000000000000401 31291211

[CR14] O’Donnell M, Maclean MJ, Sims S, Morgan VA, Leonard H, Stanley FJ (2015) Maternal mental health and risk of child protection involvement: mental health diagnoses associated with increased risk. J Epidemiol Comm Health 69:1175–1183 10.1136/jech-2014-205240 26372788

[CR15] Ross LJ, Roberts L, Derkas E, Peoples W, Bridgewater Toure P (eds) (2017) Radical reproductive justice. The Feminist Press, New York City

[CR16] Schonewille NN, Rijkers N, Berenschot A, Lijmer JG, van den Heuvel OA, Broekman BFP (2022) Psychiatric vulnerability and the risk for unintended pregnancies: a systematic review and meta-analysis. BMC Pregnancy Childbirth 22:153. 10.1186/s12884-022-04452-1 35216573 10.1186/s12884-022-04452-1 PMC8876535

[CR17] Sister Song (n.d.) Visioning New Futures for Reproductive Justice. Sister Song. Retrieved April 21, 2023, from https://www.sistersong.net/visioningnewfuturesforrj

[CR18] Swanson J, Swartz M, Ferron J, Elbogen E, Van Dorn R (2006) Psychiatric advance directives among public mental health consumers in five U.S. cities: prevalence, demand, and correlates. J Am Acad Psychiatry Law 34:43–57 16585234

[CR19] Taylor CL, Broadbent M, Mizanur K, Stewart RJ, Howard LM (2018) Predictors of severe relapse in pregnant women with psychotic or bipolar disorders. J Psychiatr Res 104:100–107. 10.1016/j.jpsychires.2018.06.019 30015264 10.1016/j.jpsychires.2018.06.019

[CR20] Trost SL, Beauregard JL, Smoots AN, Ko JY, Haight SC, Moore Simas TA, Byatt N, Madni SA, Goodman D (2021) Preventing pregnancy-related mental health deaths: insights from 14 US maternal mortality review committees, 2008–17. Health Aff 40:1551–1559. 10.1377//hlthaff.2021.00615 10.1377/hlthaff.2021.00615 PMC11135281 34606354

[CR21] Viguera AC, Whitfield T, Baldessarini RJ, Newport DJ, Stowe Z, Reminick A, Zurick A, Cohen LA (2007) Risk of recurrence in women with bipolar disorder during pregnancy: prospective study of mood stabilizer discontinuation. Am J Psychiatry 164:1817–1923. 10.1176/appi.ajp.2007.06101639 18056236 10.1176/appi.ajp.2007.06101639

[CR22] Ward HB, Fromson JA, Cooper JJ, De Oliveira G, Almeida M (2018) Recommendations for the use of ECT in pregnancy: literature review and proposed clinical protocol. Arch Womens Ment Health 21:715–722 29796968 10.1007/s00737-018-0851-0

