# Supplementary material for: Reproductive psychiatric advance directives: promoting autonomy for perinatal people with serious mental illness diagnoses
Source: Arch Womens Ment Health. 2023 Nov 10;28(3):1–6. doi: 10.1007/s00737-023-01382-5 (PMC12092564; doi:10.1007/s00737-023-01382-5)
Supplement: Supplementary file 1 — Supplementary file1 (ZIP 26 KB) [file 737_2023_1382_MOESM1_ESM.zip › Reproductive PAD Template REVISION CLEAN 10.17.23_ESM.docx]

**Reproductive Psychiatric Advance Directive**

This template is for anyone who can become pregnant. The goal is to think about and write down what your decisions about medical and psychiatric care would be if you became pregnant, but were not able to make your own decisions.

**Medical History:** The questions below help the doctors know what medical problems to look out for if you are not able to tell them yourself. The first questions are about any medical problems, and the second are about any pregnancies you may have had before.

- *Medical Problems*:
  - Do you have any of the following medical problems?
    - Diabetes?
    - High blood pressure?
    - Autoimmune disorder (such as lupus or rheumatoid arthritis?)
    - Seizure disorder?
    - Chronic kidney disease or renal failure?
    - Heart disease?
    - Asthma?
    - Spinal injuries or spinal surgery?
    - Blood clots?
    - Cancer?
  - Are there any other medication conditions you would want your doctor to know about?
- *Pregnancy History:*
  - Have you been pregnant before? (If not, skip to next section on “Medical Decisions and Informed Consent”)
  - If so, how many times?
  - Did you have any miscarriages or stillbirths?
  - Were any of your babies born early?
    - If so, how many weeks were they when they were born?
    - Did the doctor have to start labor (an induction) or did they come early on their own?
  - Did you have any C-sections?
    - If so, how many? Were they planned or an emergency?
    - Were there any problems with the C-section or recovery?
  - Did you have to have any blood transfusions?
  - Were any of your babies born with an injury, like a stuck shoulder?
  - Did you have high blood pressure or preeclampsia when pregnant?
  - Did you have a baby or child with birth defects or a genetic disorder?

**Medical Decisions and Informed Consent:**

The next questions are important in guiding health care providers in your medical care when you may not be able to make these decisions for yourself. If providers understand what matters to you as you make these decisions, they will have a better chance to make the decisions you would want. If you can, please write down what’s most important to you as you make these decisions (values, beliefs, plans, relationships, etc): ____________________________________________________________________________________________________________________________________________________________________________________________________________________________________

- *Consent for Life-Saving Procedures:*
  - Would you agree to the following procedures if it was needed to protect your life?
    - **Induction** (medication is given to start or speed up labor)
    - **C-section** (surgery to remove the baby from the mother’s belly)
    - **Vacuum or Forceps-Assisted Delivery** (using either a suction device that attaches to the baby’s head (the vacuum) or else a tool (forceps) to help the baby move through the vagina)
    - **Blood transfusion** (having someone else’s blood, that has been tested for safety, put into your body if you’ve lost too much of your own blood)
  - Would you agree to the following procedures if it was needed to protect your baby’s life?
    - **Induction**
    - **C-section**
    - **Vacuum or Forceps-Assisted Delivery**
    - **Blood transfusion**
- *Anesthesia / Pain Control for labor and postpartum:*
  - Which types of pain medications are you okay with using while in labor? What side effects are you okay with?
  - Which ones are you not okay with (for example, medicine that could be addictive)?
  - Which pain medications are you okay with taking while you are healing from labor and delivery?
- *Breastfeeding:*
  - If it is possible, would you like to nurse the baby?
  - Would you like for your baby to have formula only?
  - If it is possible, would you like for your baby to have breastmilk that is donated from someone else?

**Custody of Current Pregnancy:**

The following questions apply if there is no other legal custodian of the child, such as the father or partner. For each question, please specify your relationship to the person you are choosing and their name, phone number, email (if they have one), and address.

- If you needed someone to take care **of your baby** for a short time (like days, weeks, or months), who would you choose? Relationship: _____________________________ Contact Information: ____________________________________________________
- If you needed someone to take care of your baby for a longer period of time, who would you choose? Relationship: _______________________________________________

Contact Information: ____________________________________________________

- If you need someone to take care **of your other children** for a short time (like days, weeks or months), who would you choose? What is their contact info?
- Relationship: __________________________________________________________
- Contact Information: _____________________________________________ _______
- If you need someone to take care of your other children for a longer period of time, who would you choose? What is their contact info?
- Relationship: __________________________________________________________
- Contact Information: ____________________________________________________
- If the baby’s sibling(s) are not living with you, would you want the baby to be with its siblings?
- Is there anyone whom you would NOT want to have custody of the infant or your other children?
- Relationship: ________________________________________________________
- Contact Information: ____________________________________________________

**Family Planning / Birth Control:**

- *Family Planning:*
  - Would you like to have (more) children at some point?
  - If so, any idea when?
  - Until then, how important is it to you to prevent pregnancy?
- *Birth Control:*

There are several types of birth control. Here are some options:

- - Some are very long-lasting and work very well. These have to be inserted by a doctor either into your uterus or under the skin of your arm. They release a very small amount of hormones every day that keeps you from getting pregnant. If you decide you want another baby, a doctor can remove them and you can start trying to get pregnant again right away.
    - Would you prefer the doctor to put in an IUD in your uterus right after delivery? It lasts between 5-10 years, depending on the type.
    - Would you prefer the doctor to give you another long-acting and reversible birth control after the delivery called Nexplanon? This is a small implant that goes under the skin in your arm. It lasts around 5 years.
  - There are other types of birth control that do not stay in your body but can also be very effective if used correctly and regularly. Here are some of them:
    - Would you prefer a shot? It lasts three months, so you would need to go to the clinic every three months for a new one.
    - Would you prefer a daily pill?
    - Would you prefer a patch for your skin or ring that you put inside your vagina? Either of these need to be changed every week.
  - Would you like to discuss this further with your health care provider before deciding if you want to use birth control and if so, what type of birth control you prefer?

IMPORTANT! NONE OF THESE FORMS OF BIRTH CONTROL PREVENT SEXUALLY TRANSMITTED ILLNESSES, INCLUDING HIV OR AIDS. ONLY CONDOMS CAN DO THIS.

**Psychiatric Care in Pregnancy:**

Taking psychiatric medications in pregnancy or while breastfeeding can feel very scary because of worries that they can harm the baby. One way to make decisions about psychiatric medication in pregnancy is to think about what is the lowest risk, based on your values, preferences, and conversation with trusted providers. Some medications are higher risk and some are lower risk for the pregnancy or nursing baby. On the other hand, psychiatric symptoms can also be higher or lower risk, depending on how bad they are, how much they impact your life, and how stressful they can be. It can be helpful to think about what is lowest risk for yourself and for the baby.

- What are your current medications? Please give name, dose, when you take it, what each one does, and any side effects.
- Which symptoms are most important to manage?
- Which medications, if any, have been most helpful in managing these symptoms?
- Are there medications that you do NOT want to take, and why?
- When you are pregnant and postpartum, you will most likely have a mental health doctor and a prenatal care doctor. Would you like them to talk to each other about your care? Do you want one of them to be in charge of your care, and if so, which one?
- Is there a doctor or type of doctor that you do NOT want included in your care?
- If you have to go to to a psychiatric hospital, which hospital would you prefer?
- Are there any mental health hospitals that you do NOT want to go to?
- Is there anything else you want us to know about mental health care in pregnancy?

**Optional Questions:**

*Continuing or Ending a Pregnancy (Abortion):* Please note that by law, a health care agent or supporter cannot give permission for you to have an abortion, even if you say in your PAD that you want them to. This decision can only be made by you at a time when you are able to give informed consent. Because of this, the questions below are only for you to think and talk about with your agent or supporter if you want, but they cannot be carried out by anyone but you.

- Would you consider ending the pregnancy (getting an abortion) for any reason?
- Would you consider ending the pregnancy if it was necessary to avoid big medical problems or to save your life?
- Would you consider ending the pregnancy if you went into labor very early, around 22-24 weeks, or would you want to continue the pregnancy? This means that the fetus could possibly survive, but it might have medical problems, from mild to severe.
- Is there anyone you would definitely want to know about your decision? Anyone you would definitely NOT want to know?

*ECT (Electroconvulsive Therapy or Shock Treatment):* Please answer these questions only if you think that ECT might be a treatment you want. If not, you do not have to read or answer any further.

ECT is a procedure that can be very effective, but is usually done when other treatments, like medication, have not worked. During ECT, the patient is put to sleep with anesthesia and small electrical currents are passed through their brain for 5-10 minutes to create a seizure. While doctors do not know for sure how the seizures help, ECT can be extremely helpful for severe depression, psychosis, catatonia, and mania. The treatment is usually done 2 to 3 times a week, for 3 to 4 weeks, for a total of 6 to 12 treatments total.

Pregnant people can have ECT because does not exposure the baby to medication, and the electrical currents do not seem to harm the pregnancy or fetus. However, ECT can have side effects for the patient, such as confusion, memory loss, headache, nausea, and jaw or muscle pain. These are usually brief and can be treated with medication, but are important to know about.   Also, sometimes ECT is not available in some areas or covered by insurance.

- Would you agree to ECT if it’s needed to treat your symptoms?
- Would you like to talk about it with the doctor and think about it?
